# Supplementary material for: Attitudes towards vaccines and intention to vaccinate against COVID-19: Implications for public health communications
Source: Lancet Reg Health Eur. 2020 Dec 15;1:100012. doi: 10.1016/j.lanepe.2020.100012 (PMC7834475; doi:10.1016/j.lanepe.2020.100012)
Supplement: Supplementary file 1 [file mmc1.docx]

# **Supplementary Material**

**Table S1. Wording of study-developed items**

| **Variable** | **Question wording** | **Response options** |
| --- | --- | --- |
| Confidence in government to handle pandemic | To what extent do you have confidence in the UK Government’s ability to handle the Coronavirus situation as it continues to develop? | 1- none at all to  7- lots |
| Confidence in health system to handle pandemic | How much confidence do you have that the UK health service can cope during Covid-19? If you live in a devolved nation, we ask you to focus on the health service within your country (e.g., NHS Health Scotland / NHS Wales / HSCNI)? | 1- none at all to  7- lots |
| Compliance with COVID-19 guidelines | Are you following the recommendations from authorities to prevent spread of COVID-19? | 1- none at all to  7- very much so |
| Knowledge of COVID-19 | How would you rate your knowledge of COVID-19? | 1- very poor knowledge to  7- very good knowledge |
| Received flu vaccine last year | Did you receive an influenza (flu) vaccine last year? | Yes/no |
| Have ever refused a recommended vaccine | Have you ever refused or elected to forego a doctor-recommended vaccine for you or someone you are responsible for (e.g., your child)? | Yes/no |

**Table S2. Predictors of uncertainty and unwillingness to vaccinate against COVID-19 using two multivariable logistic regressions (weighted, *N* = 32,361)**

|  | **Undecided** | | | **Unwilling** | | |
| --- | --- | --- | --- | --- | --- | --- |
|  | **AOR** | **95% CI** | | **AOR** | **95% CI** | |
| Female (ref male) | **1·47** | 1·29 | 1·68 | **1·47** | 1·20 | 1·80 |
| Age (ref 65+) |  |  |  |  |  |  |
| 50-64 | 1·10 | 0·92 | 1·31 | 1·27 | 0·98 | 1·64 |
| 30-49 | 1·18 | 0·95 | 1·46 | **1·41** | 1·01 | 1·96 |
| 18-29 | 1·08 | 0·81 | 1·44 | 1·14 | 0·69 | 1·87 |
| Ethnic minority groups (ref White) | 1·20 | 0·94 | 1·53 | **1·73** | 1·23 | 2·42 |
| Education (ref postgraduate) |  |  |  |  |  |  |
| Undergraduate | 0·94 | 0·81 | 1·09 | 1·23 | 0·98 | 1·55 |
| A-levels or vocational | 1·06 | 0·89 | 1·27 | **1·89** | 1·46 | 2·44 |
| GCSE or lower | 1·02 | 0·84 | 1·24 | **2·39** | 1·78 | 3·19 |
| No qualifications | 1·05 | 0·78 | 1·41 | **2·47** | 1·62 | 3·77 |
| Income (ref >£90,000) |  |  |  |  |  |  |
| £60,000-£89,999 | 1·10 | 0·84 | 1·45 | 0·84 | 0·51 | 1·39 |
| £30,000-£59,999 | **1·41** | 1·11 | 1·79 | 1·21 | 0·80 | 1·83 |
| £16,000-£29,999 | **1·52** | 1·17 | 1·98 | 1·43 | 0·92 | 2·22 |
| <£16,000 | **2·14** | 1·59 | 2·89 | **2·10** | 1·29 | 3·42 |
| Employed | **1·22** | 1·03 | 1·44 | **1·31** | 1·02 | 1·68 |
| Living arrangement (ref alone) |  |  |  |  |  |  |
| With others (not children) | 1·11 | 0·94 | 1·31 | 1·22 | 0·97 | 1·53 |
| With others (including children) | **1·40** | 1·14 | 1·73 | **1·62** | 1·24 | 2·13 |
| Rural (ref urban) | 1·03 | 0·91 | 1·17 | **1·39** | 1·12 | 1·73 |
| Keyworker | **1·18** | 1·01 | 1·39 | 1·20 | 0·95 | 1·52 |
| No long-term physical health condition | **1·15** | 1·00 | 1·32 | 1·06 | 0·87 | 1·30 |
| No long-term mental health condition | 1·02 | 0·86 | 1·21 | 1·06 | 0·83 | 1·37 |
| Low confidence in government to handle pandemic | 1·00 | 0·87 | 1·15 | 1·11 | 0·89 | 1·37 |
| Low confidence in health system to handle pandemic | 1·16 | 0·99 | 1·35 | **1·29** | 1·05 | 1·59 |
| Low knowledge of COVID-19 | **1·48** | 1·27 | 1·73 | 1·17 | 0·94 | 1·47 |
| Poor compliance with COVID-19 guidelines | **1·58** | 1·37 | 1·84 | **1·69** | 1·34 | 2·13 |
| Have had COVID-19 | 1·13 | 0·97 | 1·32 | 1·15 | 0·93 | 1·44 |
| No Coronavirus Anxiety Scale symptoms | 1·15 | 0·99 | 1·35 | **1·34** | 1·05 | 1·70 |
| Mistrust of vaccine benefits (ref low) |  |  |  |  |  |  |
| Intermediate mistrust | **3·51** | 2·99 | 4·12 | **4·65** | 3·66 | 5·91 |
| High mistrust | 1·21 | 0·87 | 1·69 | **4·51** | 3·31 | 6·15 |
| Worries about unforeseen future vaccine effects (ref low) |  |  |  |  |  |  |
| Intermediate worries | **1·52** | 1·30 | 1·77 | **1·36** | 1·08 | 1·71 |
| High worries | **2·51** | 2·04 | 3·09 | **4·24** | 3·26 | 5·52 |
| Concerns about commercial profiteering (ref low) |  |  |  |  |  |  |
| Intermediate concerns | **1·91** | 1·66 | 2·22 | **1·90** | 1·51 | 2·39 |
| High level of concern | 0·94 | 0·67 | 1·32 | **1·55** | 1·07 | 2·23 |
| Preference for natural immunity (ref low) |  |  |  |  |  |  |
| Intermediate preference | **1·64** | 1·42 | 1·89 | **1·86** | 1·50 | 2·30 |
| High preference | **1·53** | 1·13 | 2·06 | **2·38** | 1·67 | 3·39 |
| Did not receive flu vaccine last year | **1·98** | 1·71 | 2·28 | **3·33** | 2·65 | 4·18 |
| Have ever refused a recommended vaccine | **1·43** | 1·17 | 1·74 | **2·56** | 1·98 | 3·30 |
| Constant | 0·02 | 0·01 | 0·03 | 0·001 | 0·0007 | 0·003 |

Note· ‘Very likely’ to vaccinate was the reference group in each of the two logistic regression models. Ethnic minority groups refers to Black, Asian and minority ethnicity. GCSE refers to General Certificate of Secondary Education. Data were weighted to the proportions of gender, age, ethnicity, country, and education obtained from the Office for National Statistics. AOR = adjusted odds ratio. CI = confidence interval.

**Table S3. Pattern of missing data in study sample (*N* = 32,361)**

|  | **Prop. missing** |
| --- | --- |
| Gender | 0 |
| Age | 0 |
| Ethnicity | 0 |
| Education | 0 |
| Income | 9·95% |
| Employment status | 0 |
| Living arrangement | 0 |
| Area of dwelling | 0 |
| Key worker status | 0 |
| Long-term physical health condition | 0 |
| Long-term mental health condition | 0 |
| Confidence in government to handle pandemic | 0·26% |
| Confidence in health system to handle pandemic | 0·25% |
| Knowledge of COVID-19 | 0·25% |
| Compliance with COVID-19 guidelines | 0·25% |
| Have had COVID-19 | 0·10% |
| Coronavirus Anxiety Symptoms scale | 19·66% |
| Prior flu vaccine behaviour | 0·56% |
| Prior vaccine refusal | 0·29% |

**Table S4. Multivariable linear regression predictors of negative attitudes towards vaccines, complete case analysis (weighted, *N* = 23,164)**

|  | **Mistrust of vaccine benefits** | | | **Worries about unforeseen future effects** | | | **Concerns about commercial profiteering** | | | **Preference for natural immunity** | | |
| --- | --- | --- | --- | --- | --- | --- | --- | --- | --- | --- | --- | --- |
|  | **Coef.** | **95% CI** | | **Coef.** | **95% CI** | | **Coef.** | **95% CI** | | **Coef.** | **95% CI** | |
| Female (ref male) | 0·02 | -0·05 | 0·10 | 0·18 | 0·11 | 0·25 | 0·04 | -0·03 | 0·11 | -0·06 | -0·12 | 0·01 |
| Age (ref 65+) |  |  |  |  |  |  |  |  |  |  |  |  |
| 50-64 | **0·18** | 0·10 | 0·27 | 0·04 | -0·03 | 0·12 | **0·20** | 0·12 | 0·28 | -0·01 | -0·09 | 0·07 |
| 30-49 | **0·21** | 0·10 | 0·32 | **-0·14** | -0·24 | -0·04 | **0·16** | 0·04 | 0·27 | **-0·24** | -0·34 | -0·13 |
| 18-29 | **-0·10** | -0·25 | 0·04 | **-0·36** | -0·53 | -0·20 | **-0·17** | -0·33 | -0·01 | **-0·65** | -0·80 | -0·51 |
| Ethnic minority groups (ref White) | **0·30** | 0·12 | 0·48 | **0·25** | 0·09 | 0·40 | **0·22** | 0·07 | 0·38 | **0·20** | 0·05 | 0·35 |
| Education (ref postgraduate) |  |  |  |  |  |  |  |  |  |  |  |  |
| Undergraduate | **0·19** | 0·12 | 0·26 | **0·27** | 0·19 | 0·34 | **0·27** | 0·20 | 0·35 | **0·35** | 0·27 | 0·42 |
| A-levels or vocational | **0·34** | 0·24 | 0·44 | **0·43** | 0·33 | 0·52 | **0·52** | 0·43 | 0·61 | **0·59** | 0·50 | 0·68 |
| GCSE or lower | **0·56** | 0·45 | 0·68 | **0·61** | 0·50 | 0·71 | **0·83** | 0·72 | 0·94 | **0·76** | 0·66 | 0·86 |
| No qualifications | **0·69** | 0·52 | 0·87 | **0·70** | 0·56 | 0·83 | **1·09** | 0·93 | 1·24 | **0·89** | 0·73 | 1·04 |
| Income (ref >£90,000) |  |  |  |  |  |  |  |  |  |  |  |  |
| £60,000-£89,999 | 0·13 | -0·03 | 0·29 | 0·12 | -0·03 | 0·27 | 0·10 | -0·01 | 0·21 | 0·09 | -0·04 | 0·22 |
| £30,000-£59,999 | **0·26** | 0·13 | 0·39 | **0·29** | 0·15 | 0·44 | **0·36** | 0·26 | 0·46 | **0·26** | 0·15 | 0·38 |
| £16,000-£29,999 | **0·39** | 0·24 | 0·53 | **0·44** | 0·27 | 0·60 | **0·56** | 0·45 | 0·67 | **0·39** | 0·26 | 0·51 |
| <£16,000 | **0·59** | 0·42 | 0·77 | **0·63** | 0·45 | 0·82 | **0·82** | 0·68 | 0·97 | **0·60** | 0·46 | 0·75 |
| Employed | 0·09 | -0·01 | 0·18 | **0·13** | 0·04 | 0·22 | 0·05 | -0·04 | 0·15 | 0·05 | -0·04 | 0·13 |
| Living arrangement |  |  |  |  |  |  |  |  |  |  |  |  |
| With others (not children) | 0·08 | -0·00 | 0·15 | 0·06 | -0·01 | 0·13 | 0·08 | -0·00 | 0·15 | **0·08** | 0·004 | 0·16 |
| With others (including children) | **0·13** | 0·01 | 0·24 | **0·19** | 0·09 | 0·29 | **0·20** | 0·09 | 0·31 | **0·18** | 0·07 | 0·28 |
| Rural (ref urban) | **0·09** | 0·02 | 0·15 | 0·06 | -0·01 | 0·13 | 0·03 | -0·03 | 0·10 | 0·05 | -0·02 | 0·11 |
| Keyworker | 0·03 | -0·07 | 0·13 | -0·00 | -0·10 | 0·09 | **0·12** | 0·03 | 0·20 | **0·09** | 0·00 | 0·18 |
| No long-term physical health condition | 0·02 | -0·05 | 0·09 | -0·01 | -0·08 | 0·06 | -0·00 | -0·07 | 0·06 | 0·05 | -0·01 | 0·12 |
| No long-term mental health condition | -0·07 | -0·16 | 0·03 | 0·04 | -0·05 | 0·14 | -0·05 | -0·15 | 0·05 | 0·05 | -0·04 | 0·14 |
| Low confidence in government to handle pandemic | 0·00 | -0·07 | 0·07 | **-0·13** | -0·19 | -0·06 | -0·06 | -0·13 | 0·01 | **-0·25** | -0·32 | -0·18 |
| Low confidence in health system to handle pandemic | **0·26** | 0·17 | 0·35 | **0·15** | 0·06 | 0·24 | **0·23** | 0·15 | 0·32 | -0·001 | -0·08 | 0·08 |
| Low knowledge of COVID-19 | **0·10** | 0·01 | 0·20 | **0·12** | 0·03 | 0·21 | **0·20** | 0·11 | 0·29 | **0·13** | 0·05 | 0·22 |
| Poor compliance with COVID-19 guidelines | **0·26** | 0·17 | 0·34 | **0·10** | 0·02 | 0·18 | **0·21** | 0·13 | 0·29 | **0·27** | 0·20 | 0·35 |
| Have had COVID-19 | **0·14** | 0·05 | 0·24 | 0·07 | -0·01 | 0·15 | **0·11** | 0·02 | 0·20 | **0·12** | 0·03 | 0·20 |
| No Coronavirus Anxiety Scale symptoms | -0·04 | -0·12 | 0·04 | **-0·07** | -0·14 | 0·01 | **-0·07** | -0·15 | -0·00 | 0·02 | -0·05 | 0·09 |
| Constant | 1·07 | 0·86 | 1·28 | 2·55 | 2·34 | 2·75 | 1·22 | 1·02 | 1·41 | 2·12 | 1·92 | 2·32 |

Note. Ethnic minority groups refers to Black, Asian and minority ethnicity. GCSE refers to General Certificate of Secondary Education. Data were weighted to the proportions of gender, age, ethnicity, country, and education obtained from the Office for National Statistics.

**Table S5. Predictors of uncertainty and unwillingness to vaccinate against COVID-19 using a multivariable multinomial regression, complete case analysis (weighted, *N* = 23,164)**

|  | **Undecided** | | | **Unwilling** | | |
| --- | --- | --- | --- | --- | --- | --- |
|  | **RRR** | **95% CI** | | **RRR** | **95% CI** | |
| Female (ref male) | **1·43** | 1·24 | 1·65 | **1·69** | 1·38 | 2·08 |
| Age (ref 65+) |  |  |  |  |  |  |
| 50-64 | 1·15 | 0·94 | 1·40 | **1·32** | 1·01 | 1·73 |
| 30-49 | 1·24 | 0·97 | 1·59 | **1·49** | 1·08 | 2·06 |
| 18-29 | 1·41 | 1·00 | 1·98 | 1·30 | 0·81 | 2·07 |
| Ethnic minority groups (ref White) | 1·09 | 0·82 | 1·45 | 1·32 | 0·88 | 1·98 |
| Education (ref postgraduate) |  |  |  |  |  |  |
| Undergraduate | 0·97 | 0·82 | 1·14 | **1·37** | 1·06 | 1·79 |
| A-levels or vocational | 1·09 | 0·89 | 1·33 | **1·82** | 1·35 | 2·45 |
| GCSE or lower | 1·20 | 0·97 | 1·48 | **2·36** | 1·70 | 3·28 |
| No qualifications | 1·16 | 0·84 | 1·62 | **2·84** | 1·79 | 4·49 |
| Income (ref >£90,000) |  |  |  |  |  |  |
| £60,000-£89,999 | 1·23 | 0·91 | 1·65 | 0·94 | 0·59 | 1·50 |
| £30,000-¬£59,999 | **1·48** | 1·17 | 1·89 | 1·20 | 0·80 | 1·81 |
| £16,000-¬£29,999 | **1·59** | 1·22 | 2·07 | 1·38 | 0·89 | 2·13 |
| <£16,000 | **2·23** | 1·67 | 2·99 | **1·91** | 1·18 | 3·08 |
| Employed | **1·26** | 1·04 | 1·51 | 1·12 | 0·85 | 1·46 |
| Living arrangement (ref live alone) |  |  |  |  |  |  |
| With others (not children) | 1·07 | 0·90 | 1·27 | 1·07 | 0·84 | 1·36 |
| With others (including children) | **1·33** | 1·06 | 1·67 | **1·51** | 1·12 | 2·04 |
| Rural (ref urban) | 1·12 | 0·97 | 1·30 | **1·38** | 1·10 | 1·71 |
| Key worker | 1·08 | 0·90 | 1·30 | 1·07 | 0·82 | 1·40 |
| No long-term physical health condition | **1·19** | 1·03 | 1·38 | 1·11 | 0·90 | 1·37 |
| No long-term mental health condition | 0·99 | 0·82 | 1·19 | 1·11 | 0·84 | 1·45 |
| Low confidence in government to handle pandemic | 0·97 | 0·83 | 1·13 | 1·15 | 0·93 | 1·43 |
| Low confidence in health system to handle pandemic | **1·29** | 1·09 | 1·53 | **1·52** | 1·20 | 1·93 |
| Low knowledge of COVID-19 | **1·33** | 1·12 | 1·59 | 1·22 | 0·95 | 1·57 |
| Poor compliance with COVID-19 guidelines | **1·50** | 1·27 | 1·76 | **2·13** | 1·70 | 2·67 |
| Have had COVID-19 | 1·08 | 0·90 | 1·29 | 1·07 | 0·84 | 1·36 |
| No Coronavirus Anxiety Scale symptoms | **1·21** | 1·04 | 1·40 | **1·38** | 1·10 | 1·74 |
| Mistrust of vaccine benefits (ref low) |  |  |  |  |  |  |
| Intermediate mistrust | **3·70** | 3·08 | 4·46 | **5·58** | 4·31 | 7·22 |
| High mistrust | 1·22 | 0·85 | 1·76 | **5·13** | 3·54 | 7·44 |
| Worries about unforeseen future vaccine effects (ref low) |  |  |  |  |  |  |
| Intermediate worries | **1·59** | 1·34 | 1·88 | **1·44** | 1·08 | 1·90 |
| High worries | **2·77** | 2·18 | 3·51 | **5·61** | 4·08 | 7·72 |
| Concerns about commercial profiteering (ref low) |  |  |  |  |  |  |
| Intermediate concerns | **1·85** | 1·56 | 2·18 | **1·50** | 1·16 | 1·95 |
| High level of concern | 0·76 | 0·53 | 1·10 | 1·10 | 0·72 | 1·68 |
| Preference for natural immunity (ref low) | **1·67** | 1·43 | 1·96 | **1·88** | 1·46 | 2·43 |
| Intermediate preference |  |  |  |  |  |  |
| High preference | **1·82** | 1·33 | 2·48 | **3·27** | 2·24 | 4·77 |
| Did not receive flu vaccine last year | **1·92** | 1·65 | 2·23 | **3·47** | 2·82 | 4·27 |
| Have ever refused a recommended vaccine | **1·39** | 1·12 | 1·73 | **2·40** | 1·85 | 3·13 |
| Constant | 0·02 | 0·01 | 0·02 | 0·001 | 0·0005 | 0·002 |

Note. Ethnic minority groups refers to Black, Asian and minority ethnicity. GCSE refers to General Certificate of Secondary Education. Data were weighted to the proportions of gender, age, ethnicity, country, and education obtained from the Office for National Statistics.

**Table S6. Unweighted and weighted descriptive statistics for negative vaccine attitudes and intent to vaccinate against COVID-19 (*N* = 32,361)**

|  | **Unweighted data** | **Weighted data** |
| --- | --- | --- |
| Variable | **Prop. or M(SE)** | **Prop. or M(SE)** |
| Negative attitudes towards vaccines |  |  |
| Mistrust of vaccine benefits | 2·02 (0·01) | 2·17 (0·02) |
| Worries about unforeseen future effects | 3·43 (0·01) | 3·53 (0·02) |
| Concerns about commercial profiteering | 2·31 (0·01) | 2·54 (0·02) |
| Preference for natural immunity | 2·80 (0·01) | 2·96 (0·02) |
| High ^a^ level of negative attitudes towards vaccines |  |  |
| Mistrust of vaccine benefits | 5·3% | 7·2% |
| Worries about unforeseen future effects | 14·6% | 16·3% |
| Concerns about commercial profiteering | 5·8% | 8·1% |
| Preference for natural immunity | 6·6% | 8·5% |
| Intermediate ^b^ level of negative attitudes towards vaccines |  |  |
| Mistrust of vaccine benefits | 14·3% | 17·2% |
| Worries about unforeseen future effects | 52·1% | 52·9% |
| Concerns about commercial profiteering | 23·9% | 28·8% |
| Preference for natural immunity | 41·8% | 44·7% |
| Low ^c^ level of negative attitudes towards vaccines |  |  |
| Mistrust of vaccine benefits | 80·4% | 75·6% |
| Worries about unforeseen future effects | 33·4% | 30·8% |
| Concerns about commercial profiteering | 70·3% | 63·1% |
| Preference for natural immunity | 51·6% | 46·7% |
|  |  |  |
| COVID-19 vaccine intentions (1= very unlikely to 6= very likely) | 4·86 (0·01) | 4·65 (0·02) |
| COVID-19 vaccine intentions |  |  |
| Very likely to vaccinate against COVID-19 | 68·9% | 63·5% |
| Undecided on COVID-19 vaccination | 20·4% | 22·5% |
| Very unlikely to vaccinate against COVID-19 | 10·7% | 14·0% |

Note. ^a^ Score of 5-6 on scale of 1-6. ^b^ Score of 3-4 on scale of 1-6. ^c^ Score of 1-2 on scale of 1-6. Data in the weighted sample were weighted to the proportions of gender, age, ethnicity, country, and education obtained from the Office for National Statistics.
